# Phylogenetic diversity (PD) and biodiversity conservation: some bioinformatics challenges

**Published:** 2007-02-17

**Authors:** Daniel P. Faith, Andrew M. Baker

**Affiliations:** 1 The Australian Museum, 6 College St., Sydney, NSW, 2010;; 2 Queensland University of Technology, School of Natural Resource Sciences, Gardens Point Campus, 2 George Street, GPO Box 2434, Brisbane, Queensland, 4001, Australia

**Keywords:** phylogenetic, biodiversity, PD, DNA barcoding, invertebrates, species problem

## Abstract

Biodiversity conservation addresses information challenges through estimations encapsulated in measures of diversity. A quantitative measure of phylogenetic diversity, “PD”, has been defined as the minimum total length of all the phylogenetic branches required to span a given set of taxa on the phylogenetic tree (Faith 1992a). While a recent paper incorrectly characterizes PD as not including information about deeper phylogenetic branches, PD applications over the past decade document the proper incorporation of shared deep branches when assessing the total PD of a set of taxa. Current PD applications to macroinvertebrate taxa in streams of New South Wales, Australia illustrate the practical importance of this definition. Phylogenetic lineages, often corresponding to new, “cryptic”, taxa, are restricted to a small number of stream localities. A recent case of human impact causing loss of taxa in one locality implies a higher PD value for another locality, because it now uniquely represents a deeper branch. This molecular-based phylogenetic pattern supports the use of DNA barcoding programs for biodiversity conservation planning. Here, PD assessments side-step the contentious use of barcoding-based “species” designations. Bio-informatics challenges include combining different phylogenetic evidence, optimization problems for conservation planning, and effective integration of phylogenetic information with environmental and socio-economic data.

## Introduction

“Biodiversity” encompasses the variety of all living forms on the planet, extending from genes to species to ecosystems (Wilson 1988). The broad nature of this definition is reflected also in its intent to capture not just known but also unknown variation. This knowledge gap extends further; not only are many components of biodiversity still unknown to science, but also the future values of components of biodiversity are hard to estimate. The study of biodiversity therefore is fundamentally about information challenges. Biodiversity conservation strategies adopt a form of risk analysis that involves estimating patterns of variation, and then trying to conserve as much of that estimated variation as possible - as a way to retain “options” (possible values) for the future.

Phylogenetic patterns among taxa (parts of the “tree of life”) summarize general patterns of variation at the level of genes or other features of taxa. Different scenarios of taxon extinctions can be expressed as potential losses in “feature diversity” and, in this way, may guide conservation priorities. Put simply, we would like to avoid “pruning” large branches from the tree of life. Viewed positively, successful conservation strategies retain as large an amount of “phylogenetic diversity” as available resources permit.

**Figure 1:**
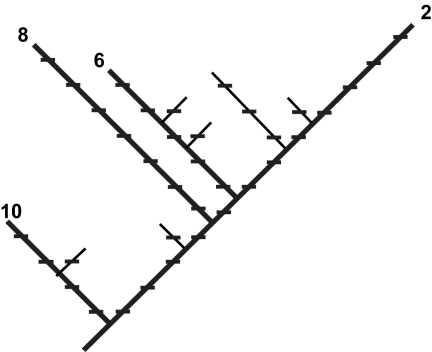
A hypothetical phylogenetic tree, redrawn from Faith 1992a. The path connecting those four taxa (2, 6, 8, and 10) having maximum expected feature diversity, is shown by the thickened lines. The number of tick marks traversed by this spanning path is 28, indicating the relative feature diversity for the set.

A quantitative measure of phylogenetic diversity, “PD”, has been defined as the minimum total length of all the phylogenetic branches required to span a given set of taxa on the phylogenetic tree (Faith 1992a). Larger PD values can be expected to correspond to greater expected feature diversity (Faith 1992b, 1994). In the example of Figure 1 (redrawn from the original figure used to define PD), the PD of the set of taxa 2, 6, 8, and 10 is 28.

Biodiversity conservation planning can focus on the PD contributions of geographic localities. When these assessments use phylogenetic patterns over several taxonomic groups, “total PD is the sum of all …branches spanned by the set of species in that area. The root of each tree is included, so even when an area has only one species from a given tree, the area still has a contribution to total PD, as indicated by the length of the spanning path from that species to the root of the tree” (Faith et al 2004).

While PD reflects “evolutionary history” (eg Faith 1994a; and branch lengths are sometimes based on time estimates), the common ancestral node or “root” used in its calculation is not extended back to the origin of all life. PD calculations are informative for comparisons and conservation planning as long as the root is taken far enough back to include all taxa under consideration in the study (for examples, see Faith 1992a, Faith 1994b; Faith et al 2004). In Fig 1, the PD of taxon 2 is 12 units, but deeper branches would be counted for broader comparisons.

**Figure 2:**
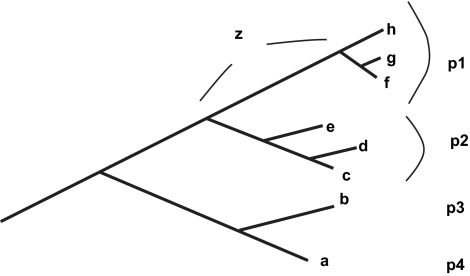
**A** phylogenetic tree example, redrawn from Faith and Williams, 2006, for taxa a through h. Taxa are found in localities p1 through p4. Taxa f, g, and h are endemic to locality p1. The PDendemism of p1 reflects the potential loss not only of the proximal connecting branches, but also the loss of the deeper branch z.

Total PD values for localities may be estimated, but more useful for conservation planning is estimation of the *additional* amounts of PD contributed by different localities, relative to some given set of localities (eg the existing protected areas in a region). In this context, “phylogenetic clumping” within a locality presents an important scenario for biodiversity conservation. Such clumping means that loss of that locality would mean loss also of the deeper phylogenetic branches linking its member taxa (dramatically illustrated in the PD analysis of global hotspots, Sechrest et al 2002). As an example, (Fig 2), if taxa f, g, and h occur uniquely in one locality (“p1”), then its loss would mean loss not only of the proximal connecting branches, but also the loss of deeper branch z (for discussion and examples, see Faith and Williams, 2006).

Such scenarios suggest that the biodiversity contribution of a locality may depend less on conventional species counts and more on the phylogenetic diversity represented. In fact, the use of PD allows one to side-step current debates about what is or is not a “species” (Faith 1992a; Mace et al 2003), and so avoid the potential over-sensitivity of planning results on species definitions (Isaac et al 2004; see also Faith &Williams 2005). Further, the use of phylogenetic pattern may better predict general biodiversity patterns in reflecting historical relationships among areas (Fig 2; Faith 1992a).

For these reasons, PD has been advocated (Faith and Williams 2005, 2006) as a way to make the best-possible use of the wealth of new data expected from large-scale DNA “barcoding” programs. This prospect raises interesting bio-informatics issues (discussed below), including how to link multiple sources of evidence for phylogenetic inference, and how to create a web-based linking of PD assessments to the barcode–of-life database (BoLD; http://www.barcoding.si.edu/index_detail.htm).

It therefore seems appropriate that an early paper in this evolutionary bio-informatics journal has addressed PD (Crozier et al 2005). Here, we will use that paper as a back-drop for our consideration of some of the bio-informatics challenges for PD applications. To facilitate this, however, we first must provide the correct definition of PD, and the useful links to the past literature, that were missing from the recent Crozier et al paper. We will outline a PD application from our own work that highlights the importance of adopting the original definition. We will then use this example to motivate discussion of some of the challenges for a PD bioinformatics.

## Characterization and application of PD

Crozier et al (2005) presented some useful example analyses using PD. However, their study sits awkwardly in the context of the extensive past literature and experience on the PD method. One awkward aspect of their paper was that it claimed to provide a first “proof of concept” for an approach that in fact was well-explored a decade ago. Crozier et al. titled their paper “Phylogenetic biodiversity assessment based on systematic nomenclature” and proposed the assessment of phylogenetic diversity based on existing taxonomy as an approach “yet to be applied to conservation biology problems”. However, this approach was taken to the “proof of concept” stage more than a decade ago, in various studies not cited in their paper. For example, the examples in Faith (1994b) provide early support for Crozier et al’s advocacy that “surrogate phylogenies can be inferred from systematic nomenclature, and these phylogenies applied in biodiversity assessment.”

The pressing bio-informatics challenge in this context continues to be, not a “proof of concept”, but an effective, practical, implementation of the approach. Recently, the Global Biodiversity Information facility (GBIF) funded a demonstration project (http://www.deh.gov.au/biodiversity/abif/bat/technical.html) showing how a PD approach, using only taxonomic information, might be implemented as web-based analyses and linked to core GBIF databases. These demonstration analyses, while promising, were restricted to calculating *total* PD estimates for localities. Future gains in practical applicability await links to the more useful PD complementarity and endemism values used in conservation planning (see above and Faith et al 2004).

**Figure 3:**
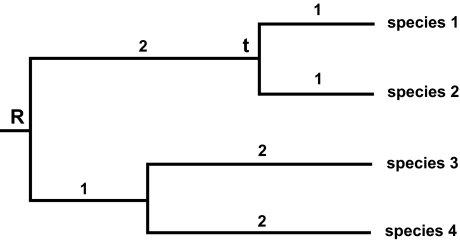
A figure re-drawn from Crozier et al (2005; figure 1), with species labeled 1 through 4. Crozier et al claim that the PD of species 1 and 2 is only 2 units, in counting branch lengths only back to node t. However, correct PD calculations in this comparative context would record the PD based on branches extending back to the shared root R, yielding a PD of 4 units for this set of two species.

The more serious omission in the Crozier et al paper is a correct characterization of the PD approach. To provide clarification, we have redrawn figure 1 from their paper (Fig 3).

Crozier et al claim that a set of taxa consisting of two species (here labeled as species 1 and species 2) would only have a PD value of 2. Surprisingly, the branches to the shared root for the *entire* group are not counted in their calculation of PD for this set. Crozier et al claim that their calculation emerges from the definition of PD, and reflects an undesirable property of the method.

Extensive description and examples of the PD method, in studies not cited by Crozier et al, counter any characterization of PD as not using the overall root for the taxa under consideration. Faith (1992a), for example, documents the practical application of PD when taxa are found in localities, and alternative sets of localities are explored. This first worked example in the literature nicely highlights the importance of taking some common root, encompassing all comparisons, into account. For set “R3” in that example (Faith (1992a, figure 3a), the corresponding limited set of taxa has a most recent common ancestor analogous to node *t* in Fig 3. Nevertheless, in the original Faith (1992a) study, the calculated PD value for the set used the deeper ancestor node common to the *entire* group of taxa under consideration, so enabling proper comparison with other sets. Thus, the total PD of the set R3 counted branches extending to this overall root branch. This carefully documented example, in the original paper defining PD, directly counters Crozier et al’s characterization of PD.

The same analysis protocol has been reflected in the later applications of PD. For example, Moritz and Faith (1998) explicitly noted that “PD values are calculated as the sum of branch lengths along the minimum spanning path (Faith 1992a) connecting all alleles from two areas and extending to the root of the tree.” Faith et al (2004) presented similar examples of PD applications. In one of their examples, when only a restricted group “a,b,c” defines a set, the PD calculation for this set nevertheless was based on the branches extending all the way back to the common root for the taxa under consideration in the study. This again directly contradicts the method for PD value assignment adopted by Crozier et al (exemplified for species 1 and 2, Fig 3).

Other PD applications over the past decade have provided re-statements of this same principle. For example, Smith et al (2000) describe their application of PD as follows:

“to estimate the underlying diversity within and among montane populations of each species, we used the (PD) measure (Faith 1992a; Faith 1994; Faith and Walker 1996; Moritz and Faith 1998). For within-region diversity, this approach sums the branch lengths in a phylogeny along the minimum path connecting all haplotypes unique to the region. For diversity spanned by combinations of regions, this approach sums the branch lengths both within and among regions and extending to the root of the tree.”

This is not to say that PD has always been applied without error. We have traced one case where, in effect, branches were double-counted, because PD values for *individual* taxa were simply added up to produce an overall score for a set of taxa (Perez-Losada et al 2002). On the other hand, another study claimed individual taxa *did not have* any possible PD values, because the overall root was not used (Barker 2002; see also Symons and Beccaloni 1999).

PD examples from the past decade or more clarify the basic properties of the PD approach. There appear to be few published examples, prior to that of Crozier et al (2005), where PD comparisons among different sets have been incorrectly made with restricted groups only evaluated back as far as their own most recent common ancestor (i.e. corresponding to the error exemplified in their figure 1). In ignoring the previous illustrations of PD calculations, Crozier et al seem to have narrowly interpreted PD as only reflecting a within-each-group variation. Of course, PD calculations do allow us to calculate the PD *unique* to a group, or even the PD of a group under the extreme assumption that no other taxa are countable (eg no other taxa are protected or otherwise selected). But these special cases of the quite general PD calculations cannot be used to characterize the overall method (indeed, specific cases of the PD calculus are given specific names such as “PD-endemism” and “PD-complementarity”; eg Faith et al 2004; Andreasen 2005).

Our clarifications help place Crozier et al’s study in the context of previous work on PD. Crozier et al criticized a “PD” method that in fact was a miss-representation of PD. They also used this argument as the rationale for defining a “new” measure, “EH”, with better properties. However, those properties turn out to be those of the true PD measure. The defined “PD” of a set of taxa (Faith 1992) is a measure reflecting its overall “evolutionary history” of divergence (eg Faith 1994a). Thus, the measure advocated and applied in their paper is *equivalent* to the long-established PD measure, but was not identified as such. We therefore recommend putting aside the discussion and the renamed measure in the Crozier et al study, in favour of retaining the characterization of PD that is well-established after more than a decade’s work.

For similar reasons, we also recommend caution in using the software referred to in their paper. The users manual (http://www.agapow.net/software/mesa) describes “phylogenetic diversity” as follows:

“this calculates the total phylogenetic distance (ie, the sum of branch lengths) over the active tree [Faith 1992a]. PD can range from 0 upwards with increasing diversity/evolutionary history. Note that as a convention, this does not include any distance on the root of the tree.”

Given this description, it appears that this software might incorrectly calculate PD, reflecting the error illustrated in Fig 3.

## How PD quantifies the biodiversity value of localities: conserving freshwater biodiversity in New South Wales, Australia

Our current applications of PD highlight both the utility of the correct definition of PD and some of the emerging bio-informatics challenges. We are exploring PD applications in an important conservation planning context in New South Wales (NSW), Australia, building on important work establishing patterns of distribution of freshwater macroinvertebrates in the Sydney water supply catchment region of south-east NSW (Baker et al 2004). Baker et al examined genetic patterns for selected aquatic macroinvertebrate genera, with a view to prioritising areas of high diversity for future conservation efforts.

**Figure 4:**
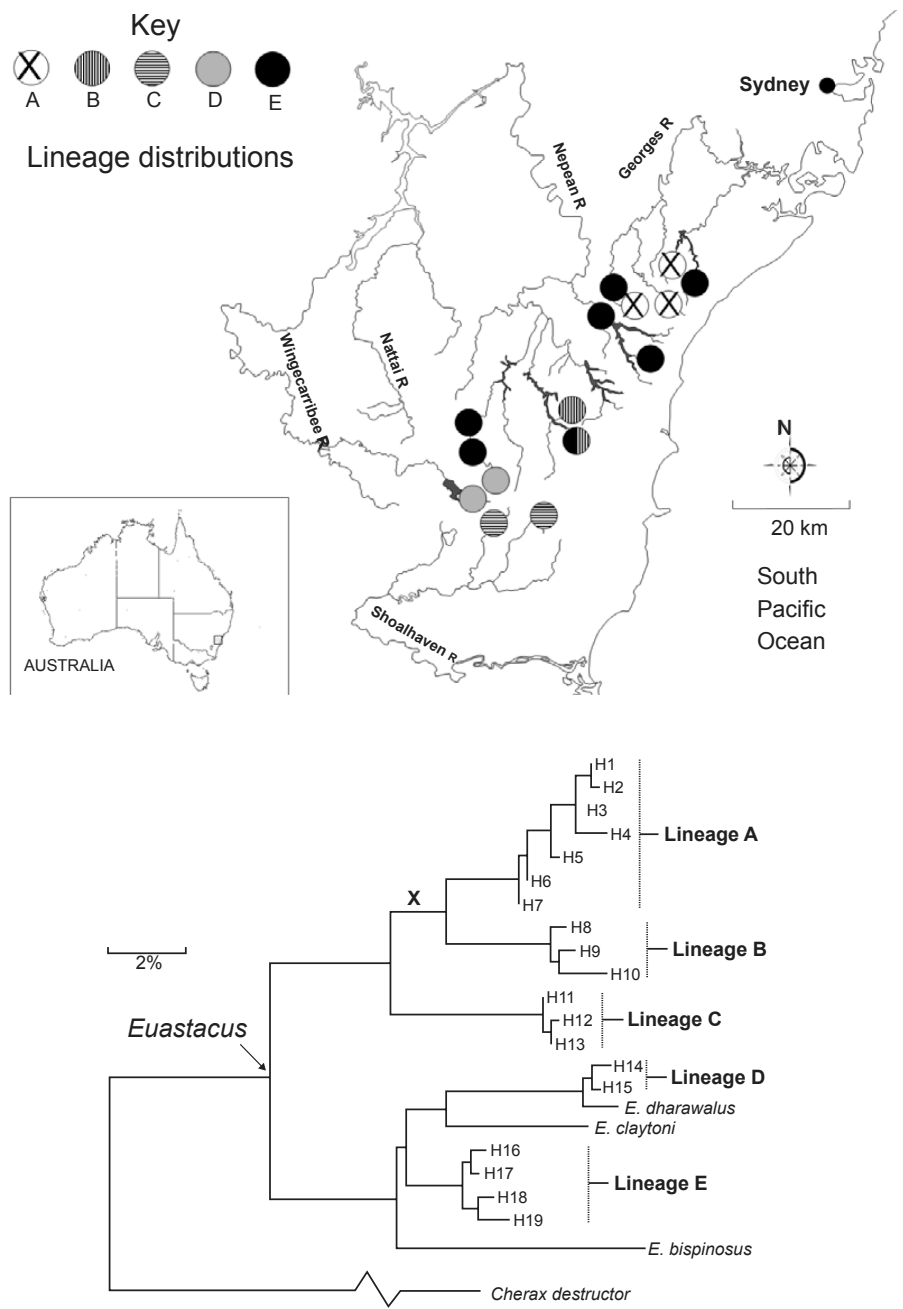
Phylogenetic and geographic distribution information for the “spiny crayfish” (*Euastacus*), as reported in Baker et al (2004) within the Sydney water supply catchment region of south-east NSW. a) The lineages labeled as A through E on the *Euastacus* phylogenetic tree shown in (b), are each represented only in a small number of places within the region. b) The phylogenetic pattern from Baker et al derived using the gene sequence, cytochrome c oxidase I gene (COI). Lineage A is a phylogenetic “sister” to lineage B. Given expected loss of biodiversity at localities containing lineage B, PD analysis assigns the localities containing lineage A higher priority, because the overall PD losses if *both* lineages were to be lost now would be high in reflecting also the loss of a shared, deeper, branch (marked **X**).

Conservation strategies in this region must respond to a number of potential threats to biodiversity. While public access to the headwater streams in the region generally is restricted, there are plans to augment sources of water supply to Sydney by constructing new dams. Further, a commercial coal seam lies beneath the headwaters of the Nepean and Georges Catchments (Fig 4a). Some mining operations currently in progress produce subsidence that could irreversibly alter drainage patterns and flow regimes–with all taxa having impacts locally, and any taxa found only in (endemic to) that locality in effect impacted “globally”.

One of the taxa of interest, with high genetic diversity in this region, is the spiny crayfish (*Euastacus*). Most *Euastacus* species have highly restricted distributions in localities that are particularly sensitive to habitat disturbance. Baker et al (2004) examined phylogenetic patterns for closely related species from the group, based on gene sequence data. They demonstrated that the group divides itself into a number of potential species (including newly discovered “cryptic” species), each of quite restricted geographic distribution. This pattern implies that different lineages on the *Euastacus* phylogenetic tree are represented only in a small number of places within the region (Fig 4a,b).

Recent events in this region highlight the utility of PD assessments for biodiversity conservation priority setting. Mining activities recently have resulted in several streams losing all surface water through cracked streambeds. This impact affected sites in the upper Nepean River where the cryptic *Euastacus* lineage B was found (Fig 4a). Our PD analysis, based on the *Euastacus* phylogenetic and distribution information, suggests a consequent higher conservation priority for another location (the upper Georges River), which at present is not impacted (but nevertheless threatened) by mining activities. This location uniquely holds lineage A (Fig 4a), a phylogenetic “sister” to lineage B. PD analysis now assigns this locality higher priority because the overall PD losses if *both* lineages were to be lost now would be high in reflecting also the loss of a shared, deeper, branch (marked X in Fig 4b). Thus, the PD-endemism value for the two localities taken together is large in reflecting this deeper branch. Note that this implied loss would not be detected if PD for the two sister taxa were wrongly calculated by counting branches back only to their most recent common ancestor (the error illustrated in Fig 3).

Our current applications of PD therefore illustrate how the assessment of phylogenetic diversity is not a matter of choosing between arbitrary definitions. Faith and Williams (2006) review other real-world applications for PD where there is some form of “phylogenetic clumping” in localities and PD calculations consequently reveal the potential loss of deeper branches.

## Issues for PD bioinformatics

A notable property of the NSW freshwater biodiversity example is that the phylogenetic information (Fig 4) was derived from a particular gene sequence, cytochrome *c* oxidase I gene (COI). New DNA “barcoding” programs for species documentation and discovery, based on COI (Hebert et al 2003) or other gene sequences, already are raising hotly-debated issues for evolutionary bio-informatics (eg, Blaxter et al 2005; Chase et al 2005; Hebert et al 2004, Moritz and Cicero 2004). PD applications suggest an important new arena for DNA-barcoding applications: the rich information source provided by large-scale barcoding can be used to address the “surrogates” problem and so provide predictions of *overall* biodiversity patterns (Faith and Williams 2005, 2006). Phylogenetic pattern sometimes is viewed as non-critical to the barcoding task of species identification (eg, Greenstone et al 2005), but phylogeny may be critical to deriving effective surrogates for general biodiversity patterns. The sensitivity of biodiversity conservation planning to species definitions suggests the possibility that the most robust information about overall biodiversity patterns from barcoding programs might be found in the associated phylogenetic patterns, rather than in the sometimes-contentious species designations (Faith and Williams 2005, 2006).

The NSW example highlights the role for PD assessments in practical biodiversity planning strategies that side-step decisions about the species-status of new, “cryptic”, variants (see also Faith and Williams 2006). The example also highlights the capacity for phylogenetic pattern to predict more general biodiversity patterns; Baker et al (2004) noted that the phylogeographic pattern for *Euastacus*, in revealing general historical relationships among localities, predicts the patterns for several other freshwater taxonomic groups. Calculated PD contributions for a given locality, based on one group (or a small number of groups), therefore may predict the more general PD contributions for that locality.

The utility of these analyses for conservation planning suggests that there is potential for a web-based PD analysis tool linked to the barcode-of life data system (see www.co1bank.uoguelph.ca/). However, practical applications for conservation planning raise special challenges relating to provision of phylogenetic information. Robust phylogenetic estimates for PD calculations require integration of the COI-based phylogenetic evidence with that from the broader “tree-of-life” databases (see http://tolweb.org/tree/phylogeny.html). Further development and evaluation of analytical methods for such combined phylogenetic analyses are needed (Bininda-Emonds 2004, Creevey and McInerney 2005). This challenge is even greater given that phylogenetic analyses will be needed for many taxonomic groups, in order to increase predictive power for overall biodiversity (for related discussion, see Soltis and Gitzendanner 1999).

This need for information over many different taxonomic groups may compete with the need for the provision of useful information for many different *places*, so that the core conservation planning task of comparative evaluation of different localities can be carried out. This raises issues about the relative cost-effectiveness of sampling more places versus using spatial predictive models, drawing on available environmental/climatic data, to extrapolate biodiversity patterns to new places (Ferrier 2002, Funk et al 2005).

In addition to the desirable integration of environmental/climatic data, our freshwater biodiversity example also highlights the need for conservation planning assessments that integrate phylogenetic/distribution information with human-use/threats information (including possible “opportunity costs” of conservation). The “bio-informatics” challenge presented by conservation planning therefore is more akin to a “multi-disciplinary-informatics”, requiring integration of biological, environmental, and socio-economic data (see also Soberón and Peterson 2004).

Conservation planning that is faced with limited resources, and/or the need to minimize conflict with non-conservation land-water use, requires new algorithms and software for determining optimal sets of localities for conservation investment. The DIVERSITY software package of Walker and Faith (1994; see also Faith and Walker 1996) incorporates PD-based measurement of biodiversity into “tradeoffs” approaches (Faith et al 1996). DIVERSITY allows identification of a set of localities in a region that not only represents its evolutionary history but also minimizes overlap with those places vulnerable to human impact. Such trade-offs are important when the representative set is to define protected areas that exclude human use.

While PD has long been integrated into systematic biodiversity conservation planning of this kind, in practice, computational challenges still exist for large problem sizes (many localities, many taxa, many adjunct criteria) and for web-based analyses. Recently, Steel (2005) has explored computational issues for PD algorithms, showing that the original “greedy” algorithm (Faith 1992a) for finding a maximum-PD set of N taxa does deliver the optimal set. Pardi and Goldman (2005) recently have extended these PD algorithms to set priorities for sequencing genomes. Future work on algorithms must address the practical need, as illustrated in the NSW example, for working with PD-complementarity and PD-endemism values, integrated with opportunity costs and other factors. Other research will explore new clustering algorithms that uncover contiguous sets of geographic units (grid cells, etc.) corresponding to centers of PD-endemism, and algorithms linking PD to new methods for extrapolation of biodiversity information to unsampled localities (Ferrier, pers. comm.).

Such phylogeny-related informatics issues for biodiversity conservation planning do not yet have a high profile. Recently, a list of research frontiers for a “museum-based informatics” (Graham et al 2004) highlighted the integration of museum collections data with phylogenetics. However, the focus was on understanding evolutionary patterns and processes (eg evolution of species “niches”), without addressing phylogenetic links to biodiversity conservation planning.

Perhaps this is why one of the first “informatics” challenges for PD-based biodiversity planning is simply the synthesis of the extensive previous work. Hopefully, ongoing critique and discussion of previous PD applications (eg Faith 2002) will help avoid one “curse” of biodiversity informatics – the accumulation of lots of variants of definitions and associated indices that, somehow, all have to be tabulated and sorted out. This ongoing task of “bio-miss-informatics” can only delay progress in addressing practical informatics problems.

## References

[b1-ebo-02-121] Andreasen K (2005). Implications of molecular systematic analyses on the conservation of rare and threatened taxa: Contrasting examples from Malvaceae. Conserv Gen.

[b2-ebo-02-121] Baker AM, Hughes JM, Dean JC (2004). Mitochondrial DNA reveals phylogenetic structuring and cryptic diversity in Australian freshwater macroinvertebrate assemblages. Mar Freshw Res.

[b3-ebo-02-121] Barker GM (2002). Phylogenetic diversity: a quantitative framework for measurement of priority and achievement in biodiversity conservation. Biol J Linn Soc.

[b4-ebo-02-121] Bininda-Emonds ORP (2004). Phylogenetic supertrees: Combining information to reveal the Tree of Life Series. Computational Biology.

[b5-ebo-02-121] Blaxter M, Mann J, Chapman T (2005). Defining operational taxonomic units using DNA barcode data. Phil Trans R Soc B.

[b6-ebo-02-121] Chase MW, Salamin N, Wilkinson M (2005). Land plants and DNA barcodes: short-term and long-term goals. Phil Trans R Soc B.

[b7-ebo-02-121] Creevey CJ, McInerney JO (2005). Clann: Investigating phylogenetic information through supertree analyses. Bioinformatics.

[b8-ebo-02-121] Crozier RH, Dunnett LJ, Agapow P (2005). Phylogenetic biodiversity assessment based on systematic nomenclature. Evolutionary Bioinformatics Online.

[b9-ebo-02-121] Faith DP (1992a). Conservation evaluation and phylogenetic diversity.. Biol. Conserv.

[b10-ebo-02-121] Faith DP (1992b). Systematics and conservation: on predicting the feature diversity of subsets of taxa. Cladistics.

[b11-ebo-02-121] Faith DP (1994a). Phylogenetic pattern and the quantification of organismal biodiversity. Phil Trans Royal Soc Lond B.

[b12-ebo-02-121] Faith DP, Forey PL, Humphries CJ, Vane-Wright RI (1994b). Phylogenetic diversity: a general framework for the prediction of feature diversity. Systematics and Conservation Evaluation.

[b13-ebo-02-121] Faith DP, Walker PA (1996). DIVERSITY.: a software package for sampling phylogenetic and environmental diversity. Reference and user’s guide. v. 2.1.

[b14-ebo-02-121] Faith DP, Williams KJ (2005). How Large-scale DNA Barcoding Programs Can. Boost Biodiversity Conservation Planning: Linking Phylogenetic Diversity (PD) Analyses to the Barcode of Life Database (BoLD). Abstract.

[b15-ebo-02-121] Faith DP, Williams KJ (2006). Phylogenetic diversity and biodiversity conservation. Yearbook of Science and Technology.

[b16-ebo-02-121] Faith DP, Walker PA, Ive J (1996). Integrating conservation and forestry production: exploring trade-offs between biodiversity and production in regional land-use assessment. For Ecol Manag.

[b17-ebo-02-121] Faith DP, Reid CAM, Hunter J (2004). Integrating Phylogenetic Diversity, Complementarity, and Endemism. Conserv Biol.

[b18-ebo-02-121] Ferrier S (2002). Mapping spatial pattern in biodiversity for regional conservation planning: where to from here. Syst Biol.

[b19-ebo-02-121] Funk VA, Richardson KS, Ferrier S (2005). Survey-gap analysis in expeditionary research: where do we go from here. Biol J Linn Soc.

[b20-ebo-02-121] Graham CH, Ferrier S, Huettman F (2004). New developments in museum-based informatics and applications in biodiversity analysis. Trends in Ecology and Evolution.

[b21-ebo-02-121] Greenstone MH, Rowley DL, Heimbach U (2005). Barcoding generalist predators by polymerase chain reaction: carabids and spiders. Mol Ecol.

[b22-ebo-02-121] Hebert PDN, Cywinska A, Ball SL (2003). Biological identifications through DNA barcodes. Proc R Soc Lond B Biol Sci.

[b23-ebo-02-121] Hebert PDN, Stoeckle MY, Zemlak TS (2004). Identification of birds through DNA barcodes. PLoS Biol.

[b24-ebo-02-121] Isaac NJB, Mallet J, Mace GM (2004). Taxonomic inflation: its influence on macroecology and conservation. Trends Ecol Evol.

[b25-ebo-02-121] Mace GM, Gittleman JL, Purvis A (2003). Preserving the Tree of Life. Science.

[b26-ebo-02-121] Moritz C, Cicero C (2004). DNA Barcoding: Promise and Pitfalls. PLoS Biol.

[b27-ebo-02-121] Moritz C, Faith DP (1998). Comparative phylogeography and the identification of genetically divergent areas for conservation. Molec Ecol.

[b28-ebo-02-121] Pardi F, Goldman N (2005). Species choice for comparative genomics: no need for cooperation. PLoS Genetics.

[b29-ebo-02-121] Perez-Losada M, Jara GC, Bond-Buckup G (2002). Conservation phylogenetics of Chilean freshwater crabs Aegla (Anomura, Aeglidae): assigning priorities for aquatic habitat protection. Biological Conservation.

[b30-ebo-02-121] Sechrest W, Brooks TM, da Fonseca GAB (2002). Hotspots and the conservation of evolutionary history. Proc Natl Acad Sci USA.

[b31-ebo-02-121] Smith TB, Holder K, Girman D (2000). Comparative avian phylogeography of Cameroon and Equatorial Guinea mountains: implications for conservation. Mol Ecol.

[b32-ebo-02-121] Soberón JM, Peterson AT (2004). Biodiversity informatics: Managing and applying primary biodiversity data. Philosophical Transactions of the Royal Society of London B.

[b33-ebo-02-121] Soltis PS, Gitzendanner MA (1999). Molecular systematics and the conservation of rare species. Conservation Biology.

[b34-ebo-02-121] Steel M (2005). Phylogenetic diversity and the greedy algorithm. Syst Biol.

[b35-ebo-02-121] Symons FB, Beccaloni GW (1999). Phylogenetic indices for measuring the diet breadths of phytophagous insects. Oecologia.

[b36-ebo-02-121] Walker PA, Faith DP (1994). DIVERSITY.-PD: Procedures for conservation evaluation based on phylogenetic diversity. Biodiv. Letters.

[b37-ebo-02-121] Wilson EO (1988). Biodiversity.

